# No evidence for the involvement of XMRV or MCV in the pathogenesis of breast cancer

**DOI:** 10.1038/bjc.2012.51

**Published:** 2012-02-16

**Authors:** G Khan, P S Philip, M Naase, K M I Al Zarouni

**Affiliations:** 1 Departments of Microbiology and Immunology, Faculty of Medicine and Health Sciences, United Arab Emirates University, Al Ain, United Arab Emirates; 2 School of Health and Social Sciences, Middlesex University, London, UK

**Keywords:** MCV, XMRV, breast cancer, prostate cancer, Merkel cell carcinoma

## Abstract

**Background:**

The aetiology of breast cancer remains elusive. A viral aetiology has been proposed, but to date no virus has been conclusively demonstrated to be involved. Recently, two new viruses, namely Merkel cell polyomavirus (MCV) and xenotropic murine leukaemia virus-related virus (XMRV) have been identified and implicated in the pathogenesis of Merkel cell carcinoma (MCC) and familial form of prostate cancer, respectively.

**Methods:**

We examined 204 samples from 58 different cases of breast cancer for presence of MCV or XMRV by PCR. Samples consisted of both malignant and non-malignant tissues. Additionally, we included 6 cases of MCC and 12 cases of prostate cancer as potential controls for MCV and XMRV, respectively.

**Results:**

All of the breast cancer samples examined were negative for both MCV and XMRV. However, 4/6 MCC and 2/12 prostate cancer samples were found to be positive for MCV and XMRV, respectively. Sequence analysis of the amplified products confirmed that these sequences belonged to MCV and XMRV.

**Conclusion:**

We conclude that there is no evidence for the involvement of MCV or XMRV in the pathogenesis of breast cancer. What role these viruses have in the pathogenesis of MCC and prostate carcinomas remains to be demonstrated.

Breast cancer is one of the most common malignancies in women worldwide. In spite of extensive research, the aetiology of this malignancy remains unknown. However, a number of risk factors have been identified, including life style, environmental and genetic factors (Veronesi *et al*, 2005). In a proportion of cases, no identifiable risk factor can be identified, prompting the idea that an oncogenic virus may be involved (Amarante and Watanabe, 2009). Indeed, several viruses have been implicated over the years (Labrecque *et al*, 1995; Bonnet *et al*, 1999; Melana *et al*, 2007; Cox *et al*, 2010; Glenn *et al*, 2010; Ariad *et al*, 2011), but none have conclusively been demonstrated to be central to the disease process (Chu *et al*, 2001; Herrmann and Niedobitek, 2003; Murray, 2006; Larrey *et al*, 2010; Khan *et al*, 2011; Silva and da Silva, 2011).

Recently, two new viruses have been identified and shown to be involved in human malignancies. The first of these is a gammaretrovirus, termed xenotropic murine leukaemia virus-related virus (XMRV) discovered in human prostate carcinomas from patients who were homozygous for the anti-viral enzyme, ribonuclease L (Urisman *et al*, 2006). If confirmed, XMRV will become the fourth member of the retroviridae family to infect humans and the second to be associated with a human malignancy (Schlaberg *et al*, 2009; Knouf *et al*, 2009; Arnold *et al*, 2010). However, the role of XMRV in prostate cancer remains controversial with a number of studies reporting negative findings (Hohn *et al*, 2009; Furuta *et al*, 2011; Stieler *et al*, 2011). Similarly, a role for XMRV in the pathogenesis of chronic fatigue syndrome was also reported (Lombardi *et al*, 2009), but this association has now been discredited and retracted (van der Meer *et al*, 2010; Paprotka *et al*, 2011; Steffen *et al*, 2011; Alberts, 2011). Furthermore, some studies have reported that XMRV is not an exogenous virus at all, but rather a mouse endogenous virus contaminant (Hue *et al*, 2010; Sato *et al*, 2010; Smith, 2010).

The other oncogenic virus that has recently been identified is the Merkel cell polyomavirus (MCV) isolated from a relatively rare form of skin cancer called Merkel cell carcinoma (MCC) (Feng *et al*, 2008). Merkel cell polyomavirus sequences have been shown to be present in up to 80% of MCCs (Feng *et al*, 2008; Garneski *et al*, 2009; Kaae *et al*, 2010). Moreover, the virus has been shown to be clonally integrated in the tumour cells and probably has a role in the pathogenesis of this malignancy. More recent studies have shown that MCV is more prevalent than initially thought and that the virus can also be detected in non-tumour tissues (Gaynor *et al*, 2007; Pastrana *et al*, 2009; Babakir-Mina *et al*, 2010; Loyo *et al*, 2010). However, in contrast to non-tumour tissue, the MCV found in MCC is not only integrated into the host cell DNA but also crucially has mutations in the viral oncogene large T (LT) antigen (Shuda *et al*, 2008), prematurely truncating the MCV LT helicase and thereby preventing autoreactivation of integrated virus replication that would be detrimental to cell survival. Similar loss of full length LT in other animal polyomaviruses has been reported (Small *et al*, 1982; Manos and Gluzman, 1984), indicating that the loss of full length LT in tumour tissues is not an experimental artefact, but probably a mechanism of polyomavirus-mediated oncogenesis (Shuda *et al*, 2008). The potential role of MCV in the pathogenesis of other human malignances, including small cell carcinoma (Wetzels *et al*, 2009), prostate cancer (Bluemn *et al*, 2009) and mesotheliomas (Bhatia *et al*, 2010), is also currently being investigated. To date, no report has been published looking at MCV and XMRV in the pathogenesis of breast cancer in a larger series of cases.

## Methods

### Clinical samples

*Breast samples* A total of 204 formalin-fixed paraffin-embedded (FFPE) breast tissues from 58 female cases of breast carcinomas were retrieved from the Department of Pathology archives after receiving ethical approval from the Al Ain Medical District Human Research Ethics Committee (application number AAMD HREC 08/39). These cases have been previously studied and further details including ER, PR and HER2 status can be found in our previous publication (Khan *et al*, 2011).

Briefly, 55/58 cases had multiple tissues (between 2 and 9, benign and malignant) that could be studied. The mean age of our cases was 48 years (median 47, range 20–97 years). Tissues consisted of:

- breast tissues: 161 samples (116 with histological evidence of malignancy, 4 benign, 41 tumour-free);
- lymph nodes: 43 samples (34 with evidence of metastasis and 9 free of malignancy).

*Prostate samples* A total of 12 FFPE cases of prostate carcinomas from the British African-Caribbean patients were available for inclusion into this study as potential positive controls for XMRV. The mean age of the patients was 71 years (median 70, range 64–84 years) with mean PSA value of 93.5 ng ml^−1^ (median 55).

*Merkel cell carcinoma samples* Six FFPE cases of MCC from Germany were included as potential positive controls for MCV. Cases consisted of four females and two males, mean age 75 years (median 75 years, range 64–87 years).

### Viral plasmid controls

A plasmid containing the entire XMRV sequence (XMRV VP62/pcDNA3) (Urisman *et al*, 2006; Dong *et al*, 2007) was obtained from Drs Robert H Silverman and Beihua Dong, through the NIH AIDS Research and Reference Reagent Program, Division of AIDS, NIAID. Another plasmid containing MCV sequence (pcDNA.MCV350 (144–3696) (Feng *et al*, 2008) was obtained from Dr Patrick Moore, also through the NIH AIDS Research and Reference Reagent Program. These plasmids were used as positive controls and to establish our PCR protocol.

### DNA extraction from clinical samples

DNA was extracted from FFPE clinical samples using standard phenol-chloroform extraction methodology previously described (Farrugia *et al*, 2010). For each sample, 4 × 5 *μ*M sections were cut and placed in a screw-cap eppendorf and DNA extracted. The quantity and purity of the extracted DNA was determined by OD260/280 ratio using the Nanodrop-1000 instrument (PeqLab Biotechnologie GmbH, Erlangen, Germany).

### PCR and sequencing

The PCR primers used for amplifying *β*-globin, XMRV and MCV have been previous described (Andres *et al*, 2010; Erlwein *et al*, 2010). Amplification was carried out using 1 U of *Taq* polymerase (Applied Biosystems Inc., Foster City, CA, USA), 0.5 mM dNTPs, 1 × PCR reaction buffer, 2 mM MgCl_2_, 6 pmol of each forward and reverse primers and 200 ng of genomic DNA template in 30 *μ*l reactions. The PCR was performed by an initial 5-min denaturation at 94 °C followed by 40 cycles of 94 °C for 60 s, 55 or 61 °C (depending on the primer set, Table 1) for 60 s and 72 °C for 60 s with a final elongation at 72 °C for 5 min. Each PCR run included a positive control and at least two negative controls. PCR reactions were carried out using an Applied Biosystems thermal cycler GeneAmp PCR System 2700. Amplified products were visualised on 2.5% agarose gel stained with ethidium bromide. All PCR amplified products clearly visible in the agarose gel were subsequently sequenced using the ABI Genetic Analyzer (3130 × 1) and the protocol of ABI Big Dye Terminator Reaction (Applied Biosystems Inc.). The sequence data were analysed using sequence analysis software v5.3 (Applied Biosystems Inc.) and compared with the reference sequences in the GenBank, accession number EF 185282.1 for XMRV and EU375803.1 for MCV.

## Results

### PCR for *β*-globin

**Figure 1:**
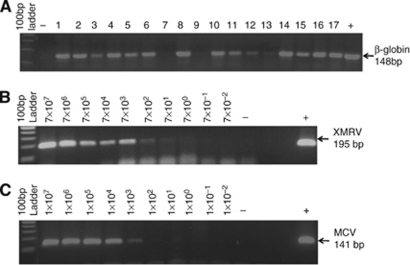
PCR for (**A**) *β*-globin, (**B**) XMRV and (**C**) MCV. DNA extracted from FFPE tissues was assessed for its amplifiable quality by performing PCR for *β*-globin. (**A**) The 148 bp PCR product (arrow) was clearly visible in agarose gel in 204 of the 219 samples tested. Samples in which *β*-globin was not amplifiable, for example, samples in lane 7 and 9, were excluded for further analysis. (**B** and **C**) Show doubling dilutions of XMRV and MCV plasmid DNA in 200 ng of cellular DNA. The 100-bp DNA ladder is also indicated.

It is well known that the quality of DNA extracted from FFPE tissues is generally poor, irrespective of the extraction methodology used (Farrugia *et al*, 2010). Extracted DNA is usually fragmented and is only suitable for amplifying small fragments, typically below 300 bp (Coates *et al*, 1991). Taking this into consideration, we employed a PCR strategy that generated products below 200 bp. Additionally, we used a ‘house-keeping gene’ (*β*-globin) to assess the amplifiable quality of the extracted DNA. DNA from a total 204 samples (from 58 cases) was amplifiable for *β*-globin (Figure 1A) and subsequently tested for XMRV and MCV. A total of 15 samples that were negative for *β*-globin were excluded from further analysis.

### PCR for XMRV and MCV using plasmid DNA

The PCR protocol for the detection of XMRV and MCV was initially optimised for sensitivity and specificity by using plasmids containing XMRV or MCV sequences serially diluted (10-fold) in 200 ng of DNA from BE(2)-M17 cell line (human neuroblastoma cell line, kind gift of Professor Omar El-Agnaf, United Arab Emirates University, UAE). We were reproducibly able to detect an estimated 700 copies of XMRV and 1000 copies of MCV DNA from 200 ng of genomic DNA (Figure 1B and C). The copy numbers were calculated using the online calculator (Staroscik, 2004). Bands from dilutions with 70 copies of XMRV and 100 copies of MCV were also visible, but were very weak. Thus, our single-round PCR method had a detection sensitivity of 70–700 copies for XMRV and 100–1000 copies for MCV.

### PCR analysis for XMRV and MCV in clinical samples

**Figure 2:**
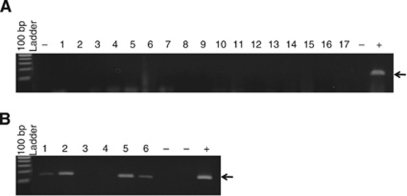
PCR for MCV in (**A**) breast and (**B**) Merkel cell carcinoma. No MCV-specific amplification product was observed in any of the 204 samples from breast cancer patients. However, 4/6 MCC were found to be positive for MCV.

The optimised PCR protocol was used for screening XMRV and MCV in breast cancer. None of the breast tissues (malignant or non-malignant) were found to be positive for XMRV or MCV (Figure 2A). Plasmid controls were consistently positive. Additionally, we examined 12 cases of prostate cancer and 6 cases of MCC as potential positive controls for XMRV and MCV, respectively. Amplification products of the expected size were visible on agarose gels for 2/12 prostate samples and 4/6 MCC samples (Figure 2B).

### Sequencing PCR amplified products

**Figure 3:**
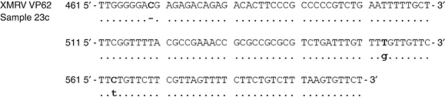
Sequence analysis of XMRV PCR product amplified from prostate sample 23c. Sequence of the region 461 to 600 nucleotides is represented and compared with the XMRV VP62 isolate (GenBank accession number: EF185282). The one deletion and two single nucleotide mutations are shown.

To confirm the identity of the PCR bands observed in the prostate and MCC samples, the PCR products were sequenced. For sequencing, sufficient DNA was available from 1/2 XMRV-positive prostate cases and 4/4 MCV-positive MCC cases. Sequence analysis confirmed the products to be of XMRV or MCV origin. The XMRV sequence amplified from the prostate case was 98% homologous to the sequence in the GenBank (accession number EF 185282.1). The prostate XMRV sequence had a single nucleotide deletion at position 469 and two single nucleotide substitutions at positions 553 and 563 (Figure 3). The MCV sequences amplified from the four MCC cases (across regions 2083–2163) were 100% homologous to the MCV strain, MKL-1 (accession number EU375803.1).

## Discussion

Breast cancer is a leading cause of death in woman worldwide and recent studies indicate that the incidence of this malignancy is increasing by approximately 3% per year (Forouzanfar *et al*, 2011). It is generally accepted that environmental factors have an important role in the aetiology of breast cancer. Of the environmental factors, viruses have received considerable attention. Indeed, a number of viruses have been implicated in the pathogenesis of breast cancer, including mouse mammary tumour virus (Fernandez-Cobo *et al*, 2006; Indik *et al*, 2007), human papillomavirus (Damin *et al*, 2004; Akil *et al*, 2008) and Epstein-Barr virus (Preciado *et al*, 2005; Mazouni *et al*, 2011). However, no known virus has yet been conclusively demonstrated to be central in the pathogenesis of this malignancy. Xenotropic murine leukaemia virus-related virus and MCV are two relatively new viruses that have been associated with human malignancies. We have examined the possibility that one of these viruses may be linked to the pathogenesis of breast cancer. We found no evidence for the involvement of these viruses. We did, however, find evidence for the presence of XMRV and MCV in a proportion of prostate and MCC cases, respectively, confirming previous findings (Urisman *et al*, 2006; Feng *et al*, 2008).

Some reports have also shown that XMRV (Lo *et al*, 2010; Fischer *et al*, 2010) and MCV (Kean *et al*, 2009; Tolstov *et al*, 2009; Pancaldi *et al*, 2011) are not restricted to tumours only and can also be found in healthy individuals and normal tissues in tumour-affected patients. Our data does not support this. We tested both malignant and non-malignant tissues, breast and lymph nodes from breast cancer patients, but failed to find viral sequences in any of the 204 samples tested. It is possible that these viruses are present in cells other than those of the breast and lymph nodes that we examined (Pancaldi *et al*, 2011). It is also possible that viral sequences are present, but at very low copy numbers (Pancaldi *et al*, 2011) and beyond the detection limit of the PCR method used in this study. We used a standard single round PCR approach rather than nested PCR, on the premises that if XMRV or MCV is involved in the pathogenesis of breast cancer then the virus would be expected to be present in all of the malignant cells and therefore easily detected by a standard single round PCR methodology. This is indeed what we found with MCV in MCC, where 4/6 cases were clearly positive for the virus. This single round PCR approach also reduces the chances of contamination and false positives.

Although, numerous studies have confirmed the association between MCV and MCC, the relation between XMRV and prostate cancer is far from clear. In fact, the very existence of XMRV as an exogenous human gammaretrovirus has been questioned (Paprotka *et al*, 2011; Knox *et al*, 2011; Cingöz *et al*, 2011). In this study, we found 2 of the 12 prostate samples to be positive for XMRV. One of the two XMRV amplified products was subsequently sequenced and clearly identified as belonging to XMRV VP62 genome. However, the sequence amplified in our case had several mutations compared to XMRV VP62 genome, suggesting that the source of XMRV in this sample was not due to contamination from plasmid XMRV VP62 used as a positive control. We had limited material from these two XMRV-positive prostate samples, and as such we were not able to confirm our findings using alternative primers targeting separate regions of XMRV. Thus, the possibility that the single nucleotide differences found in our case is due to sequencing errors cannot be excluded.

In chronic fatigue syndrome, it is now accepted that the detection of XMRV was most likely due to laboratory contamination and the original paper has now been retracted (Alberts, 2011; Cingöz *et al*, 2011; Knox *et al*, 2011; Paprotka *et al*, 2011; Steffen *et al*, 2011). Some studies have reported viral particles by electron microscopy as well as XMRV protein expression by immunohistochemistry (Schlaberg *et al*, 2009; Rodriguez and Goff, 2010; Stieler *et al*, 2010), indicating that XMRV is transcriptionally active and replication competent. From our data, we cannot, however, draw any conclusions as to whether XMRV represents endogenous or exogenous sequences. Further investigations are required to clarify this controversy and what role this virus has in the pathogenesis of prostate cancer.

## Figures and Tables

**Table 1:** Details of the PCR primers used for the amplification of XMRV, MCV and *β*-globin

**Target**	**Primer**	**Sequence**	**Location**	**Size of product**	**Annealing Temperature**
XMRV	Forward	5′-CATTCTGTATCAGTTAACCTAC-3′	411–432^a^	195	55 °C
	Reverse	5′-ATGATCTCGAGAACACTTAAAG-3′	609–588		
					
MCV	Forward	5′-GACTTTGCAAAACCATTTCCTTGA-3′	2022–045^b^	141	61 °C
	Reverse	5′-CTGCGGCTTGTTGGCAAATGG-3′	2163–143		
					
h*β*-G	Forward	5′-TGGTGGTCTACCCTTGGACC-3′	148–162^c^	148	55 °C
	Reverse	5′-GAGGTTGTCCAGGTGAGCCA-3′	296–277		

Abbreviation: H*β*-G=human *β*-globin

Location in GeneBank Accession number.

a EF 185282.1,

b EU375803.1,

c NM000518.4.
